# 394. Safety and Effectiveness of Long-Acting Cabotegravir/Rilpivirine in People with HIV and Severe Renal Impairment (CAPRI Trial)

**DOI:** 10.1093/ofid/ofaf695.132

**Published:** 2026-01-11

**Authors:** Alyssa Shon, Chiu-bin hsiao, Cassandra Oehler, Xuemei Tang, Jessica Ren, Qing Ma

**Affiliations:** University at Buffalo, Buffalo, New York; Allegheny General Hospital, Positive Health clinic, Center for Inclusion Health, AHN; Drexel University, College of Medicine, Pittsburgh, Pennsylvania; Allegheny General Hospital, Positive Health Clinic, Center for Inclusion Health, AHN, Drexel University College of Medicine, Pittsburgh, Pennsylvania; University at Buffalo, Buffalo, New York; University at Buffalo, Buffalo, New York; University at Buffalo, Buffalo, New York

## Abstract

**Background:**

Cabotegravir/Rilpivirine (CAB/RPV) represents the first and only complete long-acting injectable antiretroviral therapy. Although the unique benefits of CAB/RPV have been demonstrated in large clinical trials among people with HIV (PWH), including a favorable safety profile, few drug-drug interactions, and viral control without a daily oral pill requirement, no current evidence is available on CAB/RPV use among PWH diagnosed with chronic kidney disease (CKD).

**Figure:**
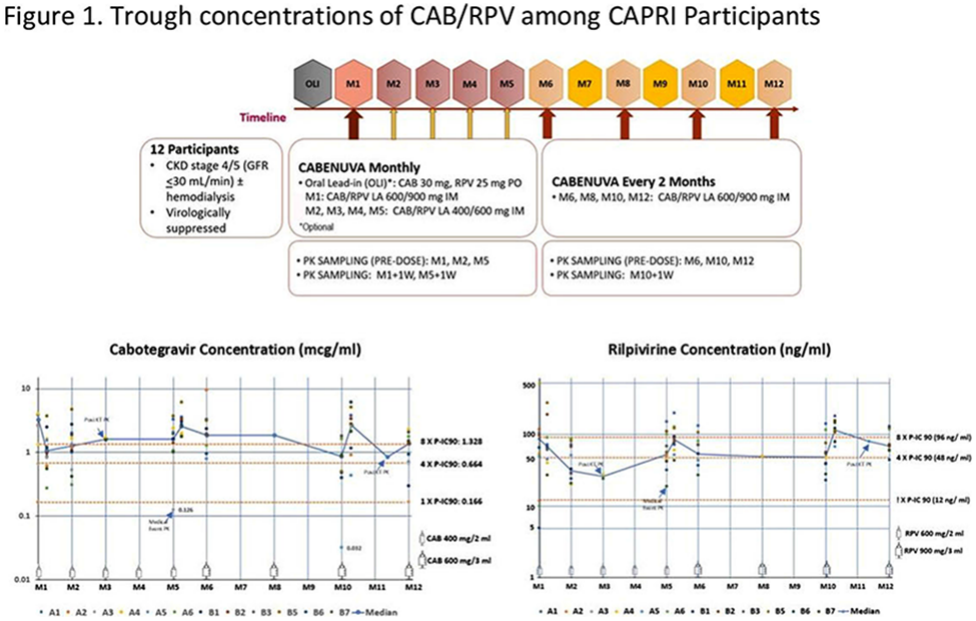

**Figure:**
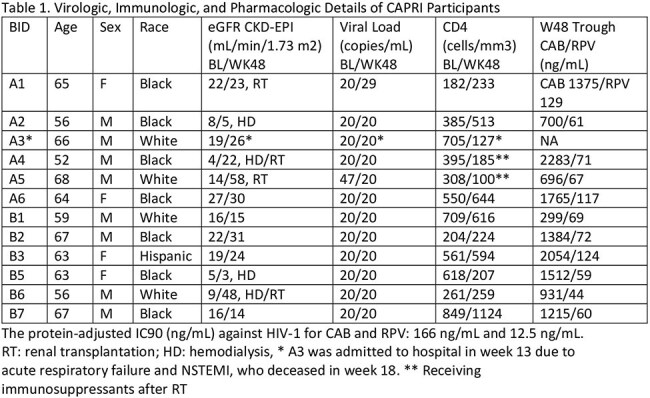

**Methods:**

A phase IV clinical trial CAPRI (NCT05601128) was designed to evaluate the effectiveness and safety of CAB/RPV LA among PWH with CKD stage 4/5 (CrCl < 30 mL/min) with or without hemodialysis. A total of 12 participants were enrolled at two clinical sites in Pittsburgh and Buffalo switching from oral therapy to CAB/RPV LA once monthly for 5 months followed by once bimonthly for 6 months. The effectiveness and safety were evaluated at the baseline and 48-week post-switch, focusing on viral load, CD4+ counts, renal function and drug concentrations.

**Results:**

CAB/RPV LA was well tolerated without major related adverse events. All participants (58% Black, 33% White, 33% female) have been virally suppressed (< 50 copies/ml) with stable CD4+ counts (mean/SD, 477/219 baseline vs 402/301 week 48). The renal function as indicated by estimated glomerular filtration rate (eGFR, CKD-EPI) remained stable (median, IQR: 16, 8-21 baseline vs 24, 14-31 week 48). CAB/RPV trough concentrations were primarily within the therapeutic ranges at week 48 (CAB mean/SD 1292/606, RPV 79/29) and the concentration-time profiles were consistent with that reported in adults with normal renal function. Virologic, immunologic, and pharmacologic details for each participant are summarized in Table 1 and changes of trough concentrations are depicted in Figure 1. No significant adverse drug reactions were reported.

**Conclusion:**

The safety and effectiveness of CAB/RPV LA has been demonstrated in CAPRI for 48 weeks, supporting its use among those with severe renal impairment. Additional advantages may include lack of adherence barriers, low drug interaction potential and infrequent dosing requirements in this population, particularly those undergoing hemodialysis.

**Disclosures:**

All Authors: No reported disclosures

